# The Psychometric Properties of the Patient Health Questionnaire-4 for Pregnant Women

**DOI:** 10.3390/ijerph17207583

**Published:** 2020-10-19

**Authors:** María de la Fe Rodríguez-Muñoz, Natalia Ruiz-Segovia, Cristina Soto-Balbuena, Huynh-Nhu Le, María Eugenia Olivares-Crespo, Nuria Izquierdo-Méndez

**Affiliations:** 1 Department of Psychology, Universidad Nacional de Educación a Distancia (UNED), 28040 Madrid, Spain; nataliaruizseg@gmail.com; 2 Deparment of Gynecology and Obstetrics, Hospital Universitario Central de Asturias, 33011 Oviedo, Spain; cristsobal@yahoo.es; 3 Department of Psychology, George Washington University, Washington, DC 20052, USA; hnle@gwu.edu; 4 Deparment of Gynecology and Obstetrics, Instituto de Salud de la Mujer José Botella Llusiá, Hospital Clínico San Carlos, Faculty of Medicine Universidad Complutense de Madrid, 28040 Madrid, Spain; meolivares@cop.es (M.E.O.-C.); nuriaizquierdo4@gmail.com (N.I.-M.)

**Keywords:** depression, anxiety, pregnancy, PHQ-4, assessment tools

## Abstract

*Background:* Perinatal anxiety and depression are common complications during pregnancy. The purpose of this study was to examine the item characteristics, reliability, validity, and factorial structure of the four-item Patient Health Questionnaire-4 (PHQ-4) and to determine the associations between scale scores and sociodemographic factors in a sample of pregnant women from Spain. *Method:* A total of 845 pregnant women were recruited from two public hospitals in Spain between 2014 and 2016. Participants completed a self-report questionnaire that included Patient Health Questionnaire-4, including the two-item Patient Health Questionnaire and the two-item Generalized Anxiety Disorder Screener. *Results:* Exploratory and confirmatory factor analysis and scale inter-correlations between the PHQ-4 and PHQ-9 revealed that the PHQ-4 has a bivariate structure and adequately assesses the dimensions of antenatal anxiety and depression. *Conclusion*: The PHQ-4 is a reliable and valid instrument to screen for depression and anxiety during pregnancy. The PHQ-4 is an ultra-brief measure that can be used to screen for antenatal depression and anxiety to prevent the negative consequences associated with these mental health conditions among mothers and infants.

## 1. Introduction

Depression is the most common complication during pregnancy [1,2]. Studies have been conducted in both clinical and non-clinical samples of pregnant women, demonstrating wide prevalence, ranging from 6.9% to 12.4% [3,4]. In Spain, the prevalence of antenatal depressive symptoms lies between 10.3% and 14.8% [5,6]. Depression is also highly comorbid with anxiety [2]. The systematic review and meta-analysis conducted by Dennis et al. (2017) [7] found that the prevalence of anxiety symptoms was 18.2% in the first-, 19.1% in the second-, and 24.6% in the third trimester of pregnancy. In Spain, Soto-Balbuena et al. (2018) [8] also found a high prevalence of anxiety during pregnancy: 19.5%, 16.8%, and 17.2% in the first-, second-, and third trimester, respectively.

Antenatal depression and anxiety have well-documented negative consequences on women and infants. Antenatal depression is associated with having a preterm birth, and low infant birthweight [9,10]. The single best predictor of major depression in the postpartum period is the presence of depressive symptoms during pregnancy [9,10,11]. Antenatal anxiety is associated with low adherence to medical advice, inadequate nutrition, and substance abuse [12]. Therefore, there is need to screen for symptoms of depression and anxiety during pregnancy to prevent these deleterious effects for women and their infants [13].

Pregnancy presents an opportunity to screen for depressive symptomatology as women are more likely to seek medical care during this period than any other period in their lives. To implement screening, it is necessary to have a good assessment tool [14]. The nine-item Patient Health Questionnaire (PHQ-9) demonstrates good psychometric properties in screening for depression during pregnancy [15]. The two-item Patient Health Questionnaire (PHQ-2), the ultra-short version of the PHQ-9, is the most validated screener for depressive symptomatology, with good criterion and convergent validity, with good sensitivity and specificity values [16]. The PHQ-2 is a psychometrically sound screening tool for major depression in adults and pregnant and postpartum women [17,18]. The seven-item Generalized Anxiety Disorder Scale (GAD-7) was validated to detect symptoms of anxiety during the antenatal period and has demonstrated high reliability and good construct validity in general population [19] and among pregnant women [10]. The two-item Generalized Anxiety Disorder Scale (GAD-2), the shortest version of the GAD-7 [20], includes the two core criteria for GAD, which have also been shown to be effective screening items for panic, social anxiety, and post-traumatic stress disorders. A validation study with primary care patients indicated good criterion validity of the GAD-2 [20].

The PHQ-2 and the GAD-2 have been combined to create a composite ultra-brief screening instrument called the Patient Health Questionnaire-4 (PHQ-4). The PHQ-4 can reliably and validly assess depressive and anxious symptomatology in the general population [21], clinical samples [22], and pregnant woman [23]. The results of the confirmatory factor analysis (CFA) revealed the best fit to be an oblique two-factor structure, which included depression and anxiety [21,24,25,26].

Some research groups have used the PHQ-4 in Spanish-speaking samples [25,26] and one with a Spanish- and English-speaking sample of pregnant women [23]. The evidence supports the validity of the two-factor PHQ-4 as a measure of anxiety and depression in the general population [2] and as a brief measure of psychological distress among pregnant women [23,27]. Its brevity is useful on clinical contexts, such as obstetric settings. However, to our knowledge, no study has used this instrument with only Spanish-speaking pregnant women.

The purpose of this study was to examine the item characteristics, reliability, validity, and factorial structure of the four-item Patient Health Questionnaire-4 (PHQ-4), and to determine the associations between scale scores and sociodemographic factors in a sample of pregnant women from Spain. We examined the item characteristics, reliability, factor structure, and construct validity of the PHQ-2, the GAD-2, and the PHQ-4. A second aim was to investigate the associations between scale scores and socio-demographic factors and to determine the relationship with the presence of anxious and depressive symptoms during pregnancy.

## 2. Materials and Methods

### 2.1. Study Population

**Figure 1:**
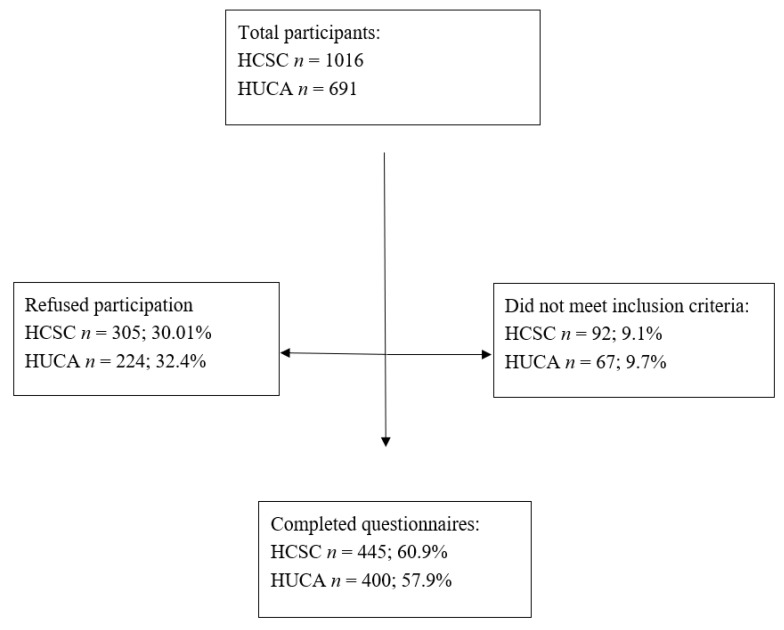
Participants. Note: HCSC = Hospital Clínico San Carlos, HUCA = Hospital Universitario Central de Asturias.

The sample included 845 pregnant women receiving antenatal care in two Spanish hospitals: San Carlos Clinic Hospital and Central University Hospital of Asturias. Inclusion criteria included receiving care at one of these hospitals beginning in their first trimester and Spanish fluency (reading, writing, speaking). Acceptance and rejection rates are presented in Figure 1. After obtaining informed consent, participants completed self-reported questionnaires on sociodemographic characteristics, depression and anxiety symptoms, and associated risk factors. This study was approved by the ethics committees from both hospitals (14/060 HOSPITAL CLINICO SAN CARLOS and 128/15 HOSPITAL CENTRAL DE ASTURIAS).

### 2.2. Data Collection and Variable Specification

Participants completed a sociodemographic questionnaire, including questions about education, employment status, marital status, illness, number of children, medication, substance use, history of medical and depression conditions.

### 2.3. Instruments

#### 2.3.1. The Patient Health Questionnaire (PHQ-2)

Whereas the PHQ-9 assesses each of the nine specific symptoms of Major Depressive Disorder (MDD) based on DSM-IV criteria, the PHQ-2 [28] includes the two core criteria for MDD: “little interest or pleasure in doing things” and “feeling down, depressed or hopeless.” The total PHQ-2 scores range from 0 to 6 [28]; scoring ≥ 2 is considered an appropriate cut-off score to detect depression during pregnancy in a sample from Spain [17].

#### 2.3.2. The Generalized Anxiety Disorder Scale (GAD-2)

The GAD-2 [21] measures two core criteria for generalized anxiety disorder, including “feeling nervous, anxious or on edge” and “not being able to stop or control worrying”. The total scores on the GAD-2 range from 0 to 6 [21]; scoring ≥ 3 indicates an appropriate cut-off point to indicate risk of developing anxiety in an English and Spanish-speaking population [26]. To date, no studies have examined the GAD-2 in Spanish-speaking sample.

#### 2.3.3. The Patient Health Questionnaire (PHQ-4)

These measures (PHQ-2 and GAD-2) were combined to form a composite four-item scale called PHQ-4 [24]. Equivalent to the parent scales, the PHQ-4 begins with the stem question: “Over the last 2 weeks, how often have you been bothered by the following problems?” Response options are “not at all”, “several days”, “more than half the days”, and “nearly every day”, scored as 0, 1, 2, and 3, respectively. The total score ranges from 0 to 12, 6 being the moderate cut-off point, and 9 the cut-off point for “red alert” for depressive-anxious symptoms in an English-speaking sample. The internal consistencies of the PHQ-4, PHQ-2 and GAD-2 were acceptable (α = 0.78, α = 0.75, and α = 0.82), respectively [21].

#### 2.3.4. Statistical Analyses Overview

The internal consistency of the PHQ-4 and its subscales were assessed to examine reliability. Specifically, item characteristics of the PHQ-4, including item means, item-intercorrelations, and corrected item-total correlations and Cronbach’s alpha without the respective item were examined.

To examine the factor structure of the PHQ-4, we conducted both exploratory and confirmatory analyses. Exploratory Factor Analyses (EFA) examined one- and two-dimensional factor constraints and were analyzed to determine the underlying individual item loadings for each model. Based upon the results of the EFA with SPSS (Version 24), using principal component solutions with varimax rotation to maximize fit, one-factor and two-factor were then analyzed using Analysis of Moment Structures (AMOS) version 24 software. In order to evaluate the dimensionality of the PHQ-4, CFAs were performed on the original two-dimensional structure of the PHQ-4 as well as a one-dimensional structure representing the PHQ-4 total score. To evaluate the fit of these structures, several indices were examined: (a) the root mean square error of approximation (RMSEA), (b) the normed fit index (NFI), (c) the comparative fit index (CFI); (d) the Akaike information criterion (AIC), (e) expected cross-validation index (ECVI), and (f) parsimonious normed fit index (PNFI).

For construct validity, we investigated the inter-correlations among the PHQ-4, GAD-2, PHQ-2, and the PHQ-9. To investigate the relationship between the sociodemographic variables and anxious and depressive symptomatology, we examined the associations between the PHQ-4, PHQ-2, and GAD-2 scores and sociodemographic characteristics (age, nationality, education level, employment and marital status and history of medical illness) that have been found to be possible risk factors for antenatal depressive and anxious symptomatology [29].

The PHQ-2, GAD-2, and PHQ-4 scores had skewed (but unimodal) distributions as dependent variables; therefore, the rank transformation of each dependent variable was performed for each model, and Bonferroni-adjustment was conducted due to multiple testing.

## 3. Results

### 3.1. Participant Characteristics

Table 1 shows the socio-demographic characteristics, lifestyle, and general health data of the pregnant women. Participants of the total sample were generally young (60%) and well educated, with most women reporting having a secondary (31.0%) or university level education (51.7%). Approximately half were first time mothers (54.2%). The majority were living with a partner (85%), employed (83.2%), and mostly native Spaniards (78.1%). Most of the participants did not report a previous history of depression (97.5%) and history of medical illness (83.2%).

Comparing the sociodemographic characteristics of women between two hospital sites (HCSC and HUCA), results showed significant differences with respect to nationality (χ^2^ = 68.2, *p* < 0.01), education level (χ^2^ = 18.2, *p* < 0.01), and marital status (χ^2^ = 8.9, *p* < 0.01). Generally, women receiving services from HUCA were more likely to be Spanish natives (90.5%) and partnered (91.9%). At HCSC, there was a higher percentage of women from other nationalities (33.1%) and with higher educational levels (52.8% university level, 34.8% secondary level). There were no significant differences between sites for age, employment status, medical illness, previous depression history, and being a first-time mother.

### 3.2. Reliability

Descriptive characteristics of the items, correlation of each item with the global scale, and reliability indices are displayed in Table 2. The two items from PHQ-2 were positively correlated (*r* = 0.52). Similarly, the two items from the GAD-2 were positively correlated (*r* = 0.60). Item-intercorrelations with items from the other subscale ranged between *r* = 0.30 and *r* = 0.49. The PHQ-2 items were positively correlated with the GAD-2 items (*r* = 0.50). All the above-mentioned correlations were significant at *p* < 0.001. The PHQ-4 total score ranges from 0 to 12, and the sample reported a score lower than the cut-off point (*M* = 2.47; *SD* = 2.40). The Cronbach’s alphas were all in the acceptable range (PHQ-4 α = 0.77, the PHQ-2 α = 0.70 and the GAD-2 α = 0.75).

### 3.3. Factor Analyses

The initial analysis of the one- and two-factor models supported the appropriateness of conducting the present EFA (Kaiser–Meyer–Olkin, KMO = 0.713, and Bartlett’s sphericity test was 922.07, *p* < 0.001).

The EFA indicated that the one-factor structure (anxiety-depression) of the PHQ-4 explained 59.63% of the total variance. In contrast, the two-factor solution explained 79.50% of the total variance. Factor 1 (i.e., anxiety) explained 59.63%, and Factor 2 (i.e., depression) explained 19.87% of the total variance. The items that loaded on Factors 1 and 2 showed acceptable internal consistency, α = 0.75 and α = 0.70, respectively. The item loadings on the corresponding factors are shown in Table 3. The depression factor was negatively associated with the anxiety factor (Spearman *r* = −0.15, *p* < 0.01).

CFA were conducted to test the one and two factor structures of the PHQ-4. The results of the CFA (Table 4) indicated excellent fit (RMSEA = 0.069, CFI = 0.99, NFI = 0.90). The value of AIC = 31.006 in the two-factor model versus the AIC = 128.127 in the one-factor model indicated greater parsimony of the two-factor structure. The expected cross-validation index (ECVI) and parsimonious normed fit index (PNFI) were evaluated with models that generated lower values and considered superior. Based on the principle of parsimony and the proportion of total explained variance, the two-factor model was determined to be superior to the one-factor model.

### 3.4. Construct Validity

The intercorrelations between the PHQ-4 and the PHQ-9 was *r* = 0.76. Similarly, the intercorrelations between the PHQ-2 and the PHQ-9 was *r* = 0.67. Finally, the intercorrelation between the GAD-2 and the PHQ-9 was *r* = 0.63. These results were all significant at *p* < 0.01, suggesting good construct validity of the PHQ-4.

### 3.5. Analysis of Variance

The independent samples *t*-test and analysis of variance (ANOVA) were used to determine whether the results of the instrument differed statistically based on participants’ demographic characteristics (Table 5). The association between the PHQ-4 and its subscale scores with demographic characteristics is shown in Table 5. Results indicate that younger women had more depressive symptoms than older women. Regarding educational level, women with more education obtained the lowest scores on the PHQ-4 compared to women with less education. Unemployed women exhibited more depressed symptoms compared to partnered women. Additionally, women who reported having a history of medical illness and smokers also report having higher levels of depressive symptoms compared to those without a history of medical illness. Similarly, participants with a history of previous depression reported having higher levels of anxious and depressive symptoms compared to those without a history of depression or tobacco use.

## 4. Discussion

The results of this study suggest that the PHQ-4 is a reliable and valid instrument that can be used to assess anxious and depressive symptoms during the antenatal period. To our knowledge, this is the first study that examined the psychometric properties and structure factor of the PHQ-4 in a sample of pregnant women in Spain.

Our first goal was to determine the psychometric characteristics of the screening measure, including the reliability, validity, and structure factor of the PHQ-4. Similar to previous studies [21,23,24,26], we used CFA to determine the most appropriate model for this population. The results provide good fit indices for the two-factor solution (anxiety, depression) of the PHQ-4. This finding is consistent with previous research conducted with samples from the general population [21,24,30] which determined that the PHQ-4 adequately assessed the dimensions of anxiety and depression. However, given that the one-factor solution explains more than half of the total variance (59.63%), the use of the total score on the PHQ-4 is justified [21]. A score greater than 6 on the PHQ-4 does not determine a diagnosis, but it does suggest the presence of anxious and/or depressive symptoms [21]. In this study, 86.1% of the pregnant women did not attain a score higher than the cut-off point on the PHQ-4.

The second goal of this study was to determine the relationship between the scale scores and the demographic characteristics of the sample. Results indicate that younger women (<25 years) had more depressive symptoms than older women (>35). These data are not consistent with a systematic review [31], which concluded that there is no consensus among researchers about the influence of age as a risk factor for antenatal depressive symptomatology. This systematic review examined psychosocial risk factors associated with antenatal depression and anxiety based on 97 international studies (the total sample was not reported). Similar to the systematic review [31], our study found that women with higher education levels obtained the lowest scores on the PHQ-4. Other studies highlight the lack of social support, low perceived levels of social support, and not having a partner as risk factors for the development of antenatal depression [28,31]. Consistent with these findings, the present study found that single women were more likely to be depressed compared to partnered women. In addition, this study found a higher prevalence of depressive symptoms among pregnant smokers, consistent with previous research [28,31]. In this study, women who reported having a history of medical illness also reported having higher levels of depressive symptoms compared to those without a history of medical illness. Similarly, pregnant women with a depression history reported having higher levels of anxious and depressive symptoms compared to those without a depression history. These findings are consistent with previous research [28,31,32].

Our study had some limitations. First, the cross-sectional design only provides information about the first trimester of pregnancy. Second, we did not perform a clinical interview to determine the construct validity of the PHQ-4, but instead, we used the PHQ-9 scores. Third, there were significant sociodemographic differences in nationality, education, marital status among pregnant women between the two hospitals. Nevertheless, results from two large hospital sites increases the generalizability of these results. Future research can adjust the cut-off points in the PHQ-4 in pregnant women because it has been observed that changes in anxious and depressive symptomatology can occur throughout the nine months of pregnancy, and longitudinal studies are important and needed in this field [17].

## 5. Conclusions

Results from our study suggest that the antenatal period provides a good opportunity to assess the mental health of women, given the regular and multiple contacts they have with the health care setting. The National Institute for Health and Care Excellence [33] in the United Kingdom and the American College of Obstetrician and Gynecologist [34] in the United States recommend screening for antenatal symptoms of depression and anxiety during routine follow-up visits. Because it is common for symptoms of anxiety and depression to be co-occur with pregnancy [35], it is essential for providers to have the necessary knowledge and tools to discriminate these symptoms adequately. In addition, given the comorbidity of depression and anxiety, the PHQ-4 could be an appropriate and short instrument to screen for both conditions. In the health care setting, providers have limited time to interact with their patients, and the PHQ-4 is a good instrument to perform a fast, reliable, and valid screening. In addition, it is important for health providers to assess sociodemographic risk factors associated with higher scores in depressive and anxious symptoms, such as smoking or a history of depression and medical illness. The PHQ-4 evaluation should also be followed-up with a more exhaustive evaluation if the pregnant women exceeds the cut-off point (>6).

Previous studies have used the PHQ-4 with Spanish-speaking samples [25,26] or Spanish- and English-speaking pregnant samples [23], but to our knowledge, this is the first study that used the PHQ-4 in a pregnant women from Spain. Our findings indicate that the PHQ-4 is a good screening tool during pregnancy that can enable appropriate follow up interventions to improve the lives of mothers and babies.

## Figures and Tables

**Table 1:** Demographic characteristics of sample.

	*n* = 845 %	HCSC (*n* = 445) *n* (%)	HUCA (*n* = 400) *n* (%)	Χ^2^	*p*-Value
Age group				1.166	0.125
*16–25 years*	8.6	41 (10.0)	29 (7.3)		
*26–36 years*	60.0	234 (56.8)	253 (63.4)		
*36–45 years*	31.3	137 (33.3)	117 (29.3)		
Nationality				68.239	0.000
*Spanish*	78.1	297 (66.9)	361 (90.5)		
*Other*	21.9	147 (33.1)	38 (9.5)		
Education level				18.163	0.000
*Primary*	17.3	55 (12.4)	91 (22.9)		
*Secondary*	31.0	155 (34.8)	106 (26.6)		
*University level*	51.7	235 (52.8)	201 (50.5)		
Employment status				1.714	0.190
*Unemployed*	25.6	105 (23.8)	110 (27.7)		
*Employed*	74.4	337 (76.2)	287 (72.3)		
Marital Status				8.891	0.003
*Single*	11.5	65 (14.6)	32 (8.1)		
*Partnered*	88.5	379 (85.4)	365 (91.9)		
History medical illness				0.321	0.571
*No*	83.2	357 (82.4)	334 (83.9)		
*Yes*	16.8	76 (14.6)	64 (16.1)		
Previous depression history				0.170	0.680
*No*	97.5	420 (97.7)	385 (97.2)		
*Yes*	2.5	10 (2.3)	11 (2.8)		
First-time mother				0.130	0.719
*No*	54.2	232 (54.4)	204 (51.1)		
*Yes*	45.8	211 (47.6)	195 (48.9)		

**Table 2:** Characteristics of the PHQ-4 items and subscales in the Spanish pregnant population.

Item	*n*	*M*	*SD*	*r^a^*	α
Depression items (PHQ-2)	824				
Little interest or pleasure in doing things		0.84	0.86	0.74 **	
Feeling down, depressed, or hopeless		0.50	0.76	0.79 **	
lePHQ-2 sum score		1.35	1.41		0.70
Anxiety items (GAD-2)	814				
Feeling nervous, anxious or on edge		0.67	0.80	0.75 **	
Not being able to stop or control worrying		0.42	0.71	0.80 **	
GAD-2 sum score		1.13	1.36		0.75
Total scale score (PHQ-4)	806	2.47	2.40		0.77

** Correlation significant at *p* < 0.01; *n* = sample size; *M* = mean; *SD* = standard deviation; *r^a^* Corrected item-total correlation; α = Cronbach’s alpha.

**Table 3:** One-factor and two-factor solution matrix of confirmatory factor analysis with varimax rotation for the PHQ-4 during pregnancy.

	One-Factor	Two-Factor	
Item	Anxiety-Depression	Factor 1-Anxiety	Factor 2-Depression
1. Little interest or pleasure in doing things	0.714		0.909
2. Feeling down, depressed, or hopeless	0.799		0.769
3. Feeling nervous, anxious or on edge	0.753	0.906	
4. Not being able to stop or control worrying	0.818	0.812	

**Table 4:** Goodness-of-fit indices for the PHQ-4 one-factor and two-factor models.

Model	*X* ^2^	*X* ^2/^ *df*	RMSEA	CFI	NFI	AIC	ECVI	PNFI
Two-factor	5.006	5.006	0.069	0.99	0.91	31.006	0.037	0.09
One-factor	104.127	52.063	0.246	0.89	0.88	128.127	0.152	0.178

Note: NFI = Normed Fit Index, CFI = Comparative Fit Index, RMSEA = Root mean square error of approximation, AIC = Akaike information criterion, ECVI = Expected Cross-Validation Index, PNFI = Parsimonious Normed Fit Index.

**Table 5:** Association of the PHQ-2, the GAD-2 and the PHQ-4 with sociodemographic characteristics.

	PHQ-2, *n* = 824		GAD-2, *n* = 814		PHQ-4, *n* = 806	
	*M* (*SD*)	Group Differences *p*-Value	*M* (*SD*)	Group Differences *p*-Value	*M* (*SD*)	Group Differences *p*-Value
Age group		0.026		0.580		0.063
*16–25*	1.82 (1.67)		1.29 (1.53)		3.15 (2.78)	
*26–35*	1.32 (1.41)		1.15 (1.36)		2.47 (2.45)	
*36–45*	1.33 (1.37)		1.09 (1.32)		2.37 (2.45)	
Education level		0.000		0.017		0.000
*Primary*	1.59 (1.61)		1.26 (1.48)		2.83 (2.66)	
*Secondary*	1.60 (1.57)		1.29 (1.45)		2.90 (2.71)	
*University*	1.14 (1.22)		1.00 (1.26)		2.12 (2.07)	
Employment status		0.049		0.234		0.074
*Unemployed*	1.53 (1.60)		1.23 (1.44)		2.75 (2.73)	
*Employed*	1.29 (1.34)		1.10 (1.33)		2.37 (2.28)	
Marital Status		0.089		0.468		0.437
*Single*	1.63 (1.67)		1.24 (1.51)		2.86 (2.72)	
*Partnered*	1.32 (1.38)		1.12 (1.34)		2.42 (2.36)	
History of medical illness		0.031		0.268		0.059
*No*	1.30 (1.34)		1.10 (1.31)		2.38 (2.24)	
*Yes*	1.63 (1.70)		1.26 (1.57)		2.89 (2.99)	
Smoking		0.029		0.47		0.046
*No*	1.37 (1.43)		1.26 (139)		2.61 (2.41)	
*Yes*	1.80 (1.73)		1.38 (1.49)		3.20 (2.86)	
Previous depression history		0.013		0.001		0.01
*No*	1.31 (1.37)		1.08 (1.30)		2.37 (2.30)	
*Yes*	2.63 (2.09)		2.78 (1.90)		5.53 (3.33)	

Group differences were examined using analysis of variance and *t*-tests. All group differences remained significant after Bonferroni-adjustment for multiple testing.
